# β1 Integrins Mediate Attachment of Mesenchymal Stem Cells to Cartilage Lesions

**DOI:** 10.1089/biores.2014.0055

**Published:** 2015-01-01

**Authors:** Daniela Zwolanek, Magdalena Flicker, Elisabeth Kirstätter, Frank Zaucke, Gerjo J.V.M. van Osch, Reinhold G. Erben

**Affiliations:** ^1^ Department of Biomedical Sciences, University of Veterinary Medicine, Vienna, Austria.; ^2^ Center for Biochemistry and Cologne Center for Musculoskeletal Biomechanics, Medical Faculty, University of Cologne, Cologne, Germany.; ^3^ Department of Orthopaedics and Otorhinolaryngology, Erasmus MC, University Medical Center Rotterdam, Rotterdam, The Netherlands.

**Keywords:** biochemistry, cellular biology, extracellular matrix, stem cells

## Abstract

Mesenchymal stem cells (MSC) may have great potential for cell-based therapies of osteoarthritis. However, after injection in the joint, only few cells adhere to defective articular cartilage and contribute to cartilage regeneration. Little is known about the molecular mechanisms of MSC attachment to defective articular cartilage. Here, we developed an *ex vivo* attachment system, using rat osteochondral explants with artificially created full-thickness cartilage defects in combination with genetically labeled MSC isolated from bone marrow of human placental alkaline phosphatase transgenic rats. Binding of MSC to full-thickness cartilage lesions was improved by serum, but not hyaluronic acid, and was dependent on the presence of divalent cations. Additional *in vitro* tests showed that rat MSC attach, in a divalent cation-dependent manner, to collagen I, collagen II, and fibronectin, but not to collagen XXII or cartilage oligomeric matrix protein (COMP). RGD peptides partially blocked the adhesion of MSC to fibronectin *in vitro* and to cartilage lesions *ex vivo*. Furthermore, the attachment of MSC to collagen I and II *in vitro* and to cartilage lesions *ex vivo* was almost completely abolished in the presence of a β1 integrin blocking antibody. In conclusion, our data suggest that attachment of MSC to *ex vivo* full-thickness cartilage lesions is almost entirely β1 integrin-mediated, whereby both RGD- and collagen-binding integrins are involved. These findings suggest a key role of integrins during MSC attachment to defective cartilage and may pave the way for improved MSC-based therapies in the future.

## Introduction

Cell therapies or cell-based gene therapies with adult multipotent mesenchymal stem cells (MSC) may have a large impact on the treatment of many diseases of various organ systems in the future.^1^ One of the typical diseases in which MSC-based therapies may hold great promise is osteoarthritis (OA). OA is the most common age-related form of arthritis with prevalence for the knee and hip joint. The initiation of the disease is triggered by genetic disposition, single major trauma, a series of microtraumas, or strenuous exercise.^2^ The few differentiated chondrocytes within the articular cartilage have only minimal regeneration potential,^3^ leading to a poor prognosis for patients suffering from OA.^4^ Murphy et al. demonstrated that the intra-articular injection of autologous bone marrow-derived MSC in a goat model of OA stimulated the regeneration of meniscal tissue and retarded progressive joint destruction.^5^ Since then, other studies investigating cartilage regeneration using MSC also reported successful and promising results in various model systems.^6–10^ However, only a small fraction of injected cells adhere to damaged cartilage and therefore contribute to tissue regeneration.^11^ In addition, it is currently a matter of debate whether the beneficial effects of MSC therapy is due to a progenitor or a nonprogenitor function of the injected cells.^12^ Therefore, the clinical potential of MSC-based therapy of OA is currently unclear.

MSC are a cell population of undifferentiated cells isolated from adult bone marrow or other tissues with the capacity to differentiate into mesodermal lineages such as bone, cartilage, muscle, or fat tissue. They can be isolated and expanded with low effort and can be differentiated into multiple cell lineages under certain culture conditions. Cultured MSC are positive for the surface markers CD44, CD73, CD90, CD105, CD106, CD120a, and CD124.^13^ Beside those, they also express various integrin subunits such as α1, α2, α3, α4, α5, α6, α7, α10, αV, αIIb, β1, β3, β4, and β5.^13–15^ Integrins are thought to play a pivotal role in adhesion of MSC to extracellular matrix (ECM) proteins and biocompatible polymers.^16–18^ The noncovalent binding of an α- and a β-subunit builds a full integrin, which is activated, and stretched in length, by binding to intracellular or extracellular proteins that drive the signaling to the ECM or to the cell.^19–21^ The integrin family consists of five different groups depending on their ligand binding. For example, collagen-binding integrins prefer the binding to the collagenous triple helix, whereas RGD-binding integrins, the largest subgroup, bind exclusively the amino acid motif Arg-Gly-Asp.^22^ Integrins mediate processes such as adhesion, migration, proliferation, cytoskeleton organization, and survival.^20^

In order to function as progenitors for cartilage regeneration intra-articularly injected MSC need to attach to articular cartilage lesions. Articular cartilage can be divided into distinct zones depending on the organization and shape of the embedded chondrocytes. The superficial zone, the outermost zone of the articular cartilage harbors only few differentiated chondrocytes with a flattened cell shape that are organized in parallel and embedded in a tight ECM.^3^ The cartilage ECM consists of high amounts of collagens and proteoglycans that build large aggregates embedded into the collagen network.^23^ The predominant collagen in articular cartilage is collagen II that forms fibrils with integrated collagen IX and XI.^24^ Noncollagenous proteins such as COMP (cartilage oligomeric matrix protein), matrilin-3, biglycan, decorin, and aggrecan, as well as the glycosaminoglycan hyaluronan, and other factors are needed to interconnect the different networks, thereby strengthening the ECM in articular cartilage.^25^ Cartilage defects result in the degradation of the ECM followed by inflammatory processes, apoptosis of chondrocytes, and subsequent loss of articular cartilage and sclerosis of the subchondral bone in combination with osteophyte formation, ultimately leading to OA.

Currently, only little is known about the molecular mechanisms involved in MSC adhesion to cartilage lesions. Here, we set out to characterize the attachment of rat MSC to full-thickness cartilage lesions and to ECM components in more detail, using a combination of *ex vivo* and *in vitro* systems. To this end, we first developed an *ex vivo* attachment system, using rat osteochondral explants with full-thickness cartilage defects in combination with genetically labeled, bone marrow-derived rat MSC. To identify the attached MSC on the tibial explants, we used human placental alkaline phosphatase (hPLAP) expressing MSC, which harbor the same characteristics as wild-type cells and are easy to track by histochemical staining.^26,27^ hPLAP is a heat-stable marker enzyme that retains its enzymatic activity after paraffin and plastic embedding.^26^ Next, we investigated the effect of adjuvants such as hyaluronic acid, serum, plasma, and divalent cations on MSC attachment to cartilage lesions. Finally, we analyzed the attachment of MSC to individual ECM proteins *in vitro*. MSC were able to bind to bone and cartilage ECM proteins via β1 integrins of the collagen-binding and RGD-binding integrin family. These findings suggest a key role of integrins during MSC attachment to defective cartilage and may pave the way for improved cell-based therapies with MSC in the future.

## Materials and Methods

### Animals

All animal experiments were approved by the ethics committees of the University of Veterinary Medicine and of the local government authorities. Wild-type and hemizygous female R26-hPLAP-tg inbred Fischer 344 rats originally generated by Kisseberth and coworkers,^28^ ubiquitously expressing the marker gene *hPLAP*, were genotyped by enzymatic histochemistry using a drop of tail blood.^28,29^ Rats were housed in groups of 2–6 at 24°C and a 12 h/12 h light/dark cycle with free access to tap water and a standard rat diet (Ssniff).

### MSC isolation

Bone marrow-derived MSC were isolated from wild-type and hPLAP-tg female rats at the age of 3–6 weeks according to the protocol described by Balmayor et al.^27^ In brief, long bones (femur, tibia, and humerus) were defleshed, cut at the epiphysis, and subsequently digested with 2.5 mg/mL collagenase type II (Invitrogen) for 2 h. The bone marrow was flushed out with complete medium containing Eagle's minimum essential medium (MEM; PAA, GE Healthcare), 10% fetal bovine serum (FBS; PAA, GE Healthcare), and 1% penicillin/streptomycin (PAA, GE Healthcare), and centrifuged. With the help of a Ficoll gradient centrifugation (Ficoll-Paque; GE Healthcare) the mononuclear cell fraction was collected, washed, and seeded in cell culture flasks (TPP; Trasadingen). After 24 h, the medium was changed and the cells were grown until 80% confluence. Cells were passaged up to passage 4. During cultivation, the medium was changed twice a week and cells were kept at 37°C with 5% CO_2_ and 3% O_2_.

### *Ex vivo* attachment assay with osteochondral explants

Tibiae from wild-type rats at the age of 5–7 months were isolated, cleaned from surrounding soft tissue without damaging the articular cartilage, and kept in PBS (without Ca^2+^ and Mg^2+^; PAA, GE Healthcare). Full-thickness defects with a diameter of 1 mm were created on the lateral and medial compartment of the tibia's articular cartilage with the help of a biopsy punch. Some tibia plateaus were left intact as a control. The tibia plateau was broken at the epiphysis, and the resulting osteochondral explants were separated into the medial and lateral compartment with a scalpel. Each explant was placed into a well of a 96-well plate (TPP; Trasadingen), washed, and kept in complete medium overnight at 37°C, 5% CO_2_, and 3% O_2_. The explants were placed into a new 96-well plate and washed 30–60 min with PBS. Single-cell suspensions of 1×10^4^ hPLAP-tg MSC per explant at passage 1–4 were incubated on upright-positioned explants for 55 min unless otherwise indicated at 37°C, 5% CO_2_, and 3% O_2_ in physiological saline (Fresenius) alone or supplemented with rat serum (native, isolated whole blood of wild-type rats) or bovine serum (FBS; PAA, GE Healthcare), plasma (native, isolated from whole blood of wild-type rats, using EDTA as anticoagulant), bone marrow plasma (native, isolated from wild-type rats), hyaluronic acid (sodium salt from *Streptococcus equi*; Sigma), divalent cations (MgCl_2_, CaCl_2_, ZnCl_2_, and MnCl_2_; Sigma), or 10 mM EDTA (Sigma).

For blocking and competition assays, saline supplemented with 50% serum and β1 integrin blocking antibody (clone Ha2/5; BD Biosciences), hamster IgM λ1 isotype control (BD Biosciences), linear RGD peptide (GRGDNP), or linear RGD control peptide (GRADSP) (both from Enzo Life Sciences) were used. The explants were washed twice with PBS to remove nonadherent cells. Adherent cells were fixed with ice-cold acetone–methanol (30:70 v/v) for 3 min at −20°C. After a washing step, the explants were placed in 1.5 mL tubes containing 1 mL PBS and incubated at 72°C and 200 rpm for 90 min to inactivate endogenous alkaline phosphatases. Subsequently, explants with attached cells were stained for heat-stable hPLAP by using TNM buffer (100 mM Tris-HCl, pH 9.5, 100 mM NaCl, 5 mM MgCl_2_) containing 0.175 mg/mL 3-bromo-4-chloro-3-indolyl phosphate (BCIP; Sigma) and 0.45 ng/mL nitrotetrazolium blue chloride (NBT; Sigma) for 3 h at room temperature (RT). The staining solution was replaced by PBS, and the explants were photographed under a stereomicroscope (Stemi SV6; Zeiss).

The obtained pictures were formatted to a size of 35 cm×35 cm and a cutout of 10 cm×15 cm with the defect in its center taken. The cutouts were analyzed with ImageJ software, and the area of attached and stained MSC was expressed as percent of the defect area. All measurements were done in triplicate with explants from three different animals per group, and repeated independently at least three times on different days with different MSC batches. Identically treated cell-free explants were used as additional negative controls.

### Histochemistry of cryosections

hPLAP-stained and quantified osteochondral explants were fixed in 70% ethanol for 48 h and decalcified with 0.5 M EDTA, pH 8.0, for 4 weeks at 4°C. Thereafter, the explants were embedded in TissueTek (Sakura Finetek), shock-frozen in liquid N_2_, and kept at −80°C until sectioning. Eleven-micrometer-thick cryosections were transferred to 3-aminopropyltriethoxy-silane (APES; Sigma)-pretreated slides. For hPLAP staining, sections were fixed with acetone–methanol (30:70 v/v) for 3 min at −20°C. As the decalcification procedure inactivates the hPLAP marker gene, reactivation had to be performed as described earlier.^30^ Therefore, all alkaline phosphatases were reactivated with 1% MgCl_2_ in 100 mM Tris-maleate buffer, pH 9.2, overnight in darkness. To inactivate endogenous alkaline phosphatases, sections were subsequently incubated at 65°C for 35 min. The staining was carried out as described in detail before.^29^ Nuclear fast red (Sigma) was used as counterstain. For Alcian blue-PAS (periodic acid-Schiff) staining, a PAS staining kit (Merck) was used according to manufacturer's instructions after sections were fixed with 4% PFA (paraformaldehyde) for 5 min at RT. Pictures of the stained sections were taken with a microscope (Axioskop 2; Zeiss).

### Immunofluorescence staining of cryosections

Anti-integrin immunofluorescence staining was performed on cryosections of hPLAP-prestained osteochondral explants as described previously.^31,32^ In brief, sections were fixed with ice-cold acetone for 2 min at −20°C and blocked with 5% normal goat serum (Vector Laboratories) in PBS supplemented with 0.2% Tween-20 for 30 min at RT. Cryosections were stained overnight at 4°C for collagen I (rabbit polyclonal; Rockland Immunochemicals), collagen II (rabbit polyclonal; Rockland Immunochemicals), α1 integrin (hamster clone Ha31/8; BD Biosciences), α2 integrin (mix of three rat clones; Emfret), and α5 integrin (rabbit polyclonal; Santa Cruz Biotechnology), or a combination of α2 integrin and collagen I or collagen II for co-immunofluorescence staining, followed by incubation with FITC-conjugated goat anti-rabbit (Sigma), Alexa 488-conjugated goat anti-rat (Molecular Probes), Texas Red-conjugated goat anti-hamster (Vector Laboratories), and Cy3-conjugated goat anti-rabbit (Jackson ImmunoResearch), either single or in mixture, for 1 h at RT. Stained sections were analyzed and pictures were taken with a microscope (Axioskop 2; Zeiss).

### *In vitro* cell attachment assay

*In vitro* cell attachment assays were carried out according to Eble et al.^33^ In brief, the following proteins were immobilized overnight at 4°C onto 96-well plates (Nunc): collagen I (bovine; Sigma), collagen II (rat, native extracted from trachea described by Dodge and Poole^34^), collagen XXII (human, gift of Manuel Koch, University of Cologne^32^), fibronectin (human; Sigma), COMP (rat, recombinant protein according to Hansen et al.^35^), and bovine serum albumin (BSA; Sigma). Free binding sites were blocked with 1% BSA in PBS for 3–4 h at 4°C. Single-cell suspensions of wild-type or hPLAP-tg MSC at passage 1–4 were incubated for 55 min at 37°C, 5% CO_2_, and 3% O_2_ in physiological saline, or MEM alone or in combination with different concentrations of divalent cations (MgCl_2_, CaCl_2_, ZnCl_2_, and MnCl_2_), 1 mM MnCl_2_ and 2 mM MgCl_2,_ or 10 mM EDTA at an average density of 6×10^4^ cells/mL. For blocking and competition assays, MEM supplemented with 1 mM MnCl_2_ and 2 mM MgCl_2_ and β1 integrin blocking antibody, hamster IgM λ1 isotype control, RGD peptide, and RGD control peptide were used. Nonadherent cells were removed by washing with PBS and adherent cells fixed with 1% glutaraldehyde (Sigma) in PBS for 15 min at RT. After staining with 0.1% (w/v), crystal violet cells were lysed with 0.2% Triton X-100 (Sigma). The amount of released dye was determined by measuring the absorption at 570 nm using a microplate reader (EnSpire; Perkin Elmer). All measurements were done in triplicate and repeated independently at least three times. Identically treated PBS-coated wells were used as blanks.

### Statistical analysis

All obtained values are reported as mean±standard deviation. All statistical analyses were performed with IBM SPSS Statistics 20 (IBM). Normal distribution of the data was analyzed by the Kolmogorov–Smirnov test. Student's *t*-test for two independent samples and one-way ANOVA followed by Student–Newman–Keuls (SNK) *post hoc* test for more than two groups were used to determine differences of indicated groups; *p*-values <0.05 were considered significant.

## Results

### Establishment of an *ex vivo* system to study MSC attachment to cartilage lesions

The attachment potential of cells can be analyzed in *in vitro* cell attachment assays. However, one disadvantage of these *in vitro* systems is that the coating with specific proteins only partially mimics the complex composition of a specific tissue. Therefore, we developed an *ex vivo* attachment system in native articular cartilage. To analyze the attachment of hPLAP-tg MSC to defective cartilage in osteochondral explants from wild-type rats, we created a full-thickness defect on the lateral and on the medial compartment of the tibia's articular cartilage, and used the defects as surface for the MSC attachment. To characterize the matrix components exposed in the defects, we performed alcian blue-PAS staining of cryosections.

**FIG. 1.**
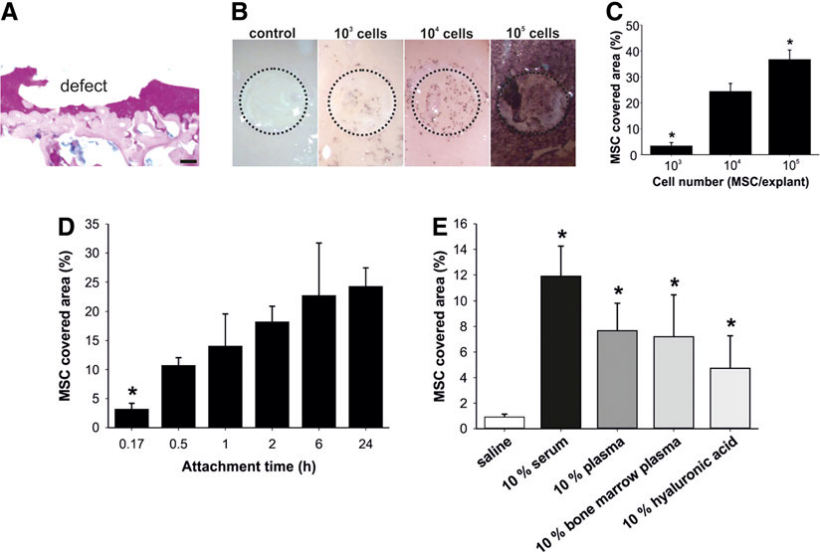

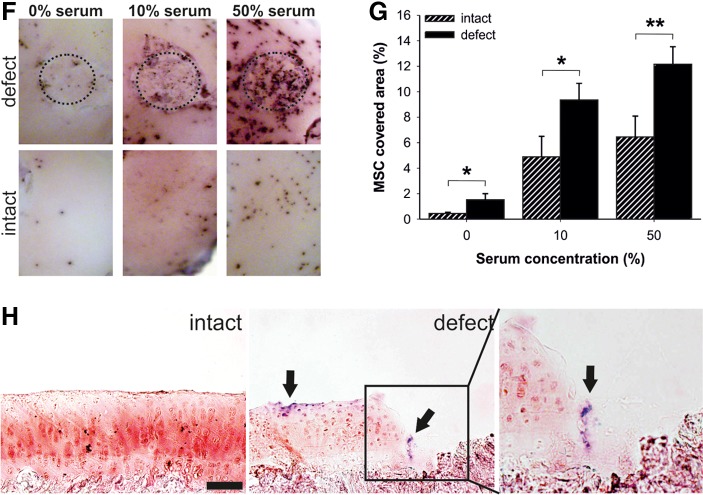
Establishment of an *ex vivo* attachment system. **(A)** Alcian blue-PAS staining of cryosections of osteochondral explants with a full-thickness defect. Articular cartilage is stained purple, subchondral bone is stained magenta, and proteoglycans and nuclei are stained blue. **(B, C)** Screen for optimal cell number. *Ex vivo* osteochondral explants from wild-type Fischer 344 rats were used to analyze the attachment of human placental alkaline phosphatase (hPLAP)-tg mesenchymal stem cells (MSC) to cartilage lesions. After attachment, the cells were fixed, and histochemically stained for hPLAP after heat inactivation of endogenous alkaline phosphatases. The stained area within the defect, corresponding to the attached cells, was quantified with the help of ImageJ software. Indicated numbers of cells suspended in 2% rat serum were allowed to attach for 24 h. Dashed circle in **(B)** indicates the full-thickness defect on the osteochondral explant. *p<0.05 vs. 10^4^ cells/explant by one-way ANOVA followed by Student–Newman–Keuls (SNK) test. **(D)** Screen for optimal attachment time. An amount of 10^4^ hPLAP-tg MSC per explant suspended in 2% rat serum were allowed to attach for the indicated time to the osteochondral explants. *p<0.05 vs. 1 h incubation time by one-way ANOVA followed by SNK test. **(E)** Testing different vehicles for MSC attachment to full-thickness cartilage defects. An amount of 10^4^ MSC suspended in saline alone or saline supplemented with 10% rat serum, plasma, bone marrow plasma, or hyaluronic acid were allowed to attach for 55 min. *p<0.05 vs. saline by one-way ANOVA followed by SNK test. **(F, G)** Influence of increasing concentrations of bovine serum to MSC attachment. An amount of 10^4^ MSC suspended in saline supplemented with 0–50% serum were allowed to attach to osteochondral explants for 55 min. Attachment to intact and defective surfaces was quantified on separate explants. Dashed circle in **(F)** indicates the full-thickness defect on the osteochondral explant. *p<0.05 and **p<0.001 by Student's *t*-test. Data represent mean values±SD (*n*≥3). All experiments were performed in triplicate and in at least three independent experiments. **(H)** Histochemical hPLAP/nuclear fast red staining of osteochondral explants. hPLAP-tg MSC (arrows) were stained in dark violet on cryosections of intact and defective osteochondral explants. Cartilage is stained orange-red and subchondral bone is stained red. Scale bar=100 μm.

Figure 1A shows that hyaline cartilage, calcified cartilage, as well as subchondral bone were exposed in the full-thickness defects. Single-cell suspensions of donor MSC isolated from hPLAP-tg rats were allowed to attach to the osteochondral explants. The area covered with hPLAP-tg MSC within the defect was quantified after washing, fixation, and histochemical staining of the hPLAP-tg cells. We first sought to optimize the seeding cell density in this osteochondral explants (Fig. 1B,C). When seeding 10^4^ cells per explant, we reached an optimal readout with single cells being distributed homogenously over the defect area, and room for quantification of increases and decreases in cell attachment. The initial attachment of cells is followed by processes such as spreading, proliferation, and/or migration.^36^ Therefore, we next determined the optimal adhesion time for our *ex vivo* attachment assay. Most of the seeded hPLAP-tg MSC attached within 0.5–1 h to the defective cartilage (Fig. 1D). After 2 h of incubation, only a small further increase in the covered area could be observed. It is likely that this further increase after prolonged incubation for up to 2 h was caused by spreading during which the cells get flattened and increase their contact surface. Taken together, we established a novel *ex vivo* system based on the attachment of hPLAP-tg MSC to cartilage lesions on osteochondral explants. This system is similar to the classic *in vitro* attachment assay but with the advantage of using native tissue as attachment surface.

### Serum and divalent cations facilitate MSC adhesion to defective cartilage

Many studies describe the use of serum as vehicle for MSC application in cell-based therapies.^6,10^ In addition, the use of hyaluronic acid is described to have a positive effect on MSC attachment after intra-articular injection.^5,8^ We therefore tested several vehicles suitable for increasing MSC adhesion to cartilage lesions in our osteochondral explant system. Administration of hPLAP-tg MSC in 10% rat serum enhanced the attachment compared to saline, as well as 10% of plasma or bone marrow plasma (Fig. 1E). The use of 10% hyaluronic acid also increased MSC attachment, but to a lower extent than serum, plasma, or bone marrow plasma. A more detailed analysis showed that the addition of serum dose-dependently increased the MSC-covered area on both the intact and the defective cartilage, but that adhesion to the defective cartilage was significantly higher at all serum concentrations at the used seeding density of 10^4^ cells per explant (Fig. 1F,G). For the quantification of MSC attachment on intact explants, independent explants without defect were used. It is interesting to note in Figure 1F that on explants with a defect, more MSC attached to the surrounding intact cartilage surface compared to intact explants without a defect. This suggests that already minor damage (also seen in Fig. 1H, middle panel, left arrow) of the cartilage surface in the surroundings of the full-thickness defect induced by handling and cleaning of the sample facilitates attachment of MSC. Histological staining of hPLAP-tg MSC in cryosections confirmed the attachment of these cells in and in the surroundings of the full-thickness defects in the osteochondral explants (Fig. 1H). These results show that serum enhances MSC adhesion to cartilage lesions.

**FIG. 2.**
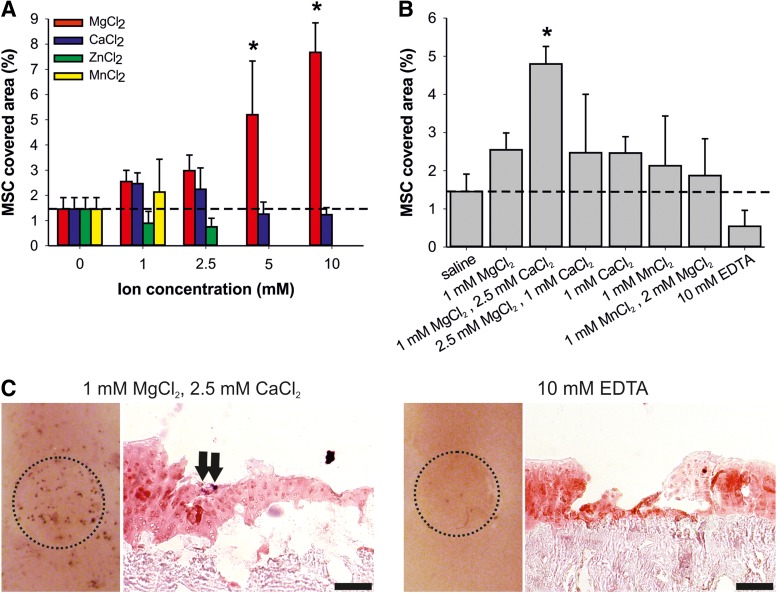
Divalent cations are necessary for *ex vivo* MSC attachment to cartilage lesions. **(A, B)** Influence of different concentrations **(A)** and combinations **(B)** of divalent cations on MSC attachment to cartilage defects. MSC suspended in saline were preincubated with the indicated divalent cation concentrations or with 10 mM EDTA, and allowed to attach to osteochondral explants for 55 min. Only attachment to the cartilage defects was quantified. Data represent mean values±SD (*n*≥3). All experiments were performed in triplicate and in at least three independent experiments. Note that for Mn^2+^, only the 1 mM concentration and for Zn^2+^, only the 1 mM and 2.5 mM concentrations were tested. *p<0.05 vs. saline by one-way ANOVA followed by SNK test. **(C)** Horizontal images and cryosections of hPLAP-stained osteochondral explants seeded with hPLAP-tg MSC in Mg^2+^ and Ca^2+^-supplemented saline or EDTA. Left panels show representative horizontal images of hPLAP-stained osteochondral explants used for quantification in **(B)**. Dashed circle indicates the full-thickness defect on the osteochondral explant. In hPLAP-stained cryosections, attached hPLAP-tg MSC (arrows) were found in the defects after incubation in saline supplemented with 1 mM Mg^2+^ and 2.5 mM Ca^2+^ (left), but not when suspended in 10 mM EDTA (right). Nuclear fast red was used as counterstaining. Cartilage is stained orange-red and subchondral bone is stained red. Scale bar=100 μm.

Earlier studies reported positive effects of magnesium and calcium on cell attachment.^37–39^ We therefore tested the ability of divalent cations such as magnesium, calcium, zinc, and manganese to improve the adhesion of MSC to defective cartilage in our system. Magnesium caused a dose-dependent increase in the MSC-covered area up to 10 mM concentrations, whereas calcium and manganese showed only a nonsignificant enhancement at 1 and 2.5 mM (Fig. 2A). Surprisingly, zinc, which is known to have an impact on the binding affinity and conformation of ECM proteins in cartilage,^40^ had no effect on MSC attachment compared with saline as vehicle. When using combinations of divalent cations as present in MEM medium, a nearly 2-fold increase in MSC adhesion was observed with a near-physiological combination of 1 mM magnesium and 2.5 mM calcium as compared to single components and other ion combinations (Fig. 2B). In addition to saline, the divalent cation chelator EDTA^21^ was used as a negative control. Horizontal images of hPLAP-stained explants and histological staining of attached MSC confirmed these findings (Fig. 2C). In conclusion, we found that serum and the divalent cations magnesium and calcium improved the adhesion of MSC to full-thickness cartilage defects in our explant system.

### Collagen I, collagen II, and fibronectin mediate MSC attachment *in vitro*

**FIG. 3.**
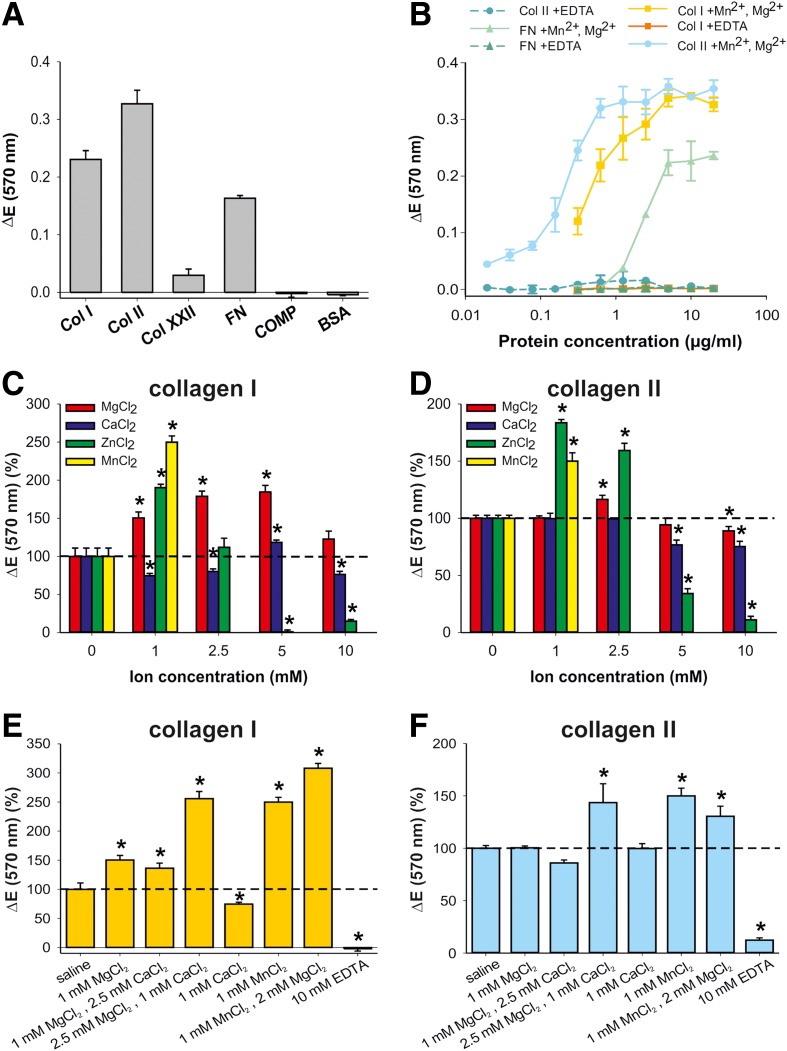
*In vitro* attachment of MSC to extracellular matrix (ECM) proteins of bone and cartilage. **(A)** Screen of MSC adhesion to abundant ECM proteins. An amount of 20 μg/mL of protein was immobilized at 4°C overnight. **(B)** Adhesion of MSC to collagen I, collagen II, and fibronectin. Serial dilutions of collagen I (Col I, 0.3–20 μg/mL), collagen II (Col II, 0.02–20 μg/mL), and fibronectin (FN, 0.3–20 μg/mL) were immobilized at 4°C overnight. In **(A)** and **(B)**, MSC suspended in MEM were allowed to attach to the substrate for 55 min in the presence of Mn^2+^ and Mg^2+^ or EDTA, followed by crystal violet staining. Relative attachment is presented as Δ*E*, measured extinction minus nonspecific attachment to PBS-pretreated wells. Data represent mean values±SD. (*n*=3). At least three independent assays were performed for all experiments. Bovine serum albumin (BSA) was used as negative control. **(C, D)** Influence of different concentrations of divalent cations on MSC attachment to collagen I and II. An amount of 2.5 μg/mL collagen I **(C)** or collagen II **(D)** was immobilized onto 96-well plates overnight. **(E, F)** Influence of combinations of divalent cations on the attachment of MSC. An amount of 2.5 μg/mL collagen I **(E)** or collagen II **(F)** was immobilized overnight. In **(C)**–**(F)**, MSC suspended in saline were preincubated with the indicated divalent cation concentrations or with 10 mM EDTA, and were allowed to attach to the substrate for 55 min. Relative attachment is presented as relative Δ*E*, normalized to the attachment (Δ*E*) of MSC suspended in saline. Data are mean±SD (*n*=3). At least three independent assays were performed for all experiments. Note that for Mn^2+^, only the 1 mM concentration was measured. *p<0.05 vs. saline by one-way ANOVA followed by SNK test.

To evaluate the specific matrix components mediating MSC attachment to full-thickness cartilage lesions, we performed *in vitro* cell attachment assays with prominent ECM proteins of cartilage and bone. In a one-point measurement to screen for putative interaction partners, we used the following ECM proteins: collagen I, present in bone, and the major cartilage proteins collagen II, collagen XXII, COMP, and fibronectin, which is known to be expressed in bone and cartilage. Only collagen I, collagen II, and fibronectin mediated MSC attachment. MSC adhered neither to collagen XXII, a prominent marker of the articular cartilage surface,^31^ nor to COMP (Fig. 3A). Collagen I, collagen II, and fibronectin mediated the MSC attachment in a concentration-dependent and saturable manner in the presence of the divalent cations manganese and magnesium (Fig. 3B). MSC adhesion to collagen I, collagen II, and fibronectin was inhibited completely by 10 mM EDTA (Fig. 3B).

To compare *ex vivo* and *in vitro* experiments, we tested several divalent cations for their ability to influence MSC attachment *in vitro*. In general, all tested cations had their strongest positive influence on MSC adhesion at physiological concentrations of 1 or 2.5 mM. Zinc of 1 mM had the most pronounced effect on MSC adhesion to collagen II (Fig. 3D). Zinc is present at a concentration of about 1 mM in cartilage and has the ability to change the conformation of ECM proteins such as collagen II, collagen IX, and COMP, and influences their binding ability to each other.^40^ Interestingly, calcium had a negative effect on the adhesion to collagen I and no effect on the adhesion to collagen II.

In addition, we tested combinations of divalent cations, similar to the above-mentioned *ex vivo* experiments. Combinations of 1 mM manganese with 2 mM magnesium and of 2.5 mM magnesium with 1 mM calcium had the most positive effects on MSC attachment to collagen I (Fig. 3E). The adhesion of MSC to collagen II was significantly increased when using 1 mM manganese with 2 mM magnesium, or 2.5 mM magnesium with 1 mM calcium (Fig. 3F). In agreement with the experiments shown in Figures 2B and 3B, the attachment of MSC to collagen I and collagen II was almost completely inhibited in the presence of 10 mM EDTA (Fig. 3E,F). Collectively, these data demonstrate that MSC are able to attach, in a divalent cation-dependent manner, to the ECM proteins collagen I, collagen II, and fibronectin, all present in bone and cartilage tissue.

### β1 integrins mediate MSC attachment *in vitro* and *ex vivo*

Because the MSC attachment was almost completely abolished in the presence of EDTA, we hypothesized that integrins may be involved in the adhesion of MSC to matrix proteins exposed in cartilage lesions. MSC are known to express the β1 integrin subunit on their cell surface,^13,14^ which is also used as a marker to identify MSC after isolation. Furthermore, they express the subunits α1, α2, and α10, belonging to the family of collagen-binding integrins, and the subunits α5 and αV belonging to the RGD-binding integrins.^14,15^ Thus, MSC express both collagen- and RGD-binding integrins.

**FIG. 4.**
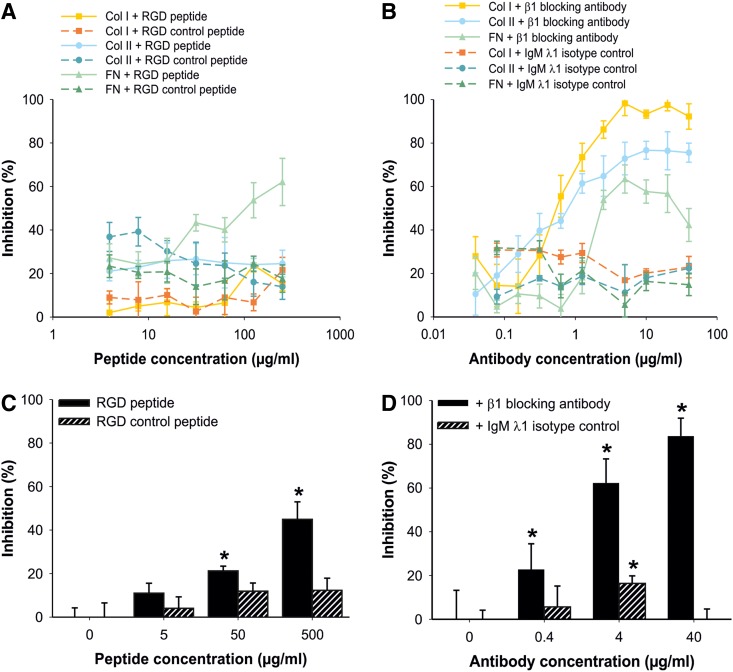
Adhesion of MSC *in vitro* and *ex vivo* is β1 integrin dependent. **(A, B)** Influence of RGD peptides and a β1 integrin blocking antibody on MSC attachment to ECM proteins *in vitro*. An amount of 2.5 μg/mL of collagen I (Col I), collagen II (Col II), and fibronectin (FN) was immobilized at 4°C overnight. MSC were suspended in MEM containing Mn^2+^ and Mg^2+^, supplemented with the indicated concentrations of RGD peptide, RGD control peptide, β1 integrin blocking antibody, or IgM λ1 isotype control, and were allowed to attach for 55 min to the substrate. Attachment was quantified as Δ*E*, measured extinction minus nonspecific attachment to PBS-pretreated wells, and is given as % inhibition. Data represent mean values±SD (*n*=3). At least three independent assays were performed for all experiments. **(C, D)** Influence of RGD peptides and a β1 integrin blocking antibody on MSC attachment to full-thickness cartilage lesions *ex vivo*. MSC were suspended in saline containing 50% bovine serum and supplemented with the indicated concentrations of RGD peptide, RGD control peptide, β1 integrin blocking antibody, or IgM λ1 isotype control. MSC were allowed to attach for 55 min to the osteochondral explants. Only attachment to the cartilage defects was quantified. Data represent mean values±SD (*n*≥3). All experiments were performed in triplicate and in at least three independent experiments. *p<0.05 vs. 50% bovine serum without blocking supplement by one-way ANOVA followed by SNK test.

To identify the integrins involved in MSC adhesion to ECM proteins and cartilage lesions, we performed competition and inhibition assays. We first performed competition experiments with RGD peptide in the *in vitro* attachment system. Only the MSC attachment to fibronectin could be inhibited by about 60% by the RGD peptide (Fig. 4A). The attachment to collagen I and collagen II was unaffected by the RGD peptide (Fig. 4A). An RGD control peptide served as a negative control. Blocking assays with a monoclonal β1 integrin blocking antibody revealed that the β1 integrin subunit was essential for binding of MSC to collagen I and collagen II. If the β1 integrin subunit was blocked, MSC could no longer adhere to collagen I and collagen II, whereas addition of an isotype control antibody had no influence on the attachment of MSC (Fig. 4B). In contrast, attachment of MSC to fibronectin was not completely blocked by the β1 integrin blocking antibody (Fig. 4B). These results demonstrate that MSC bind to fibronectin via RGD-binding integrins, and to collagen I and collagen II via collagen-binding integrins.

To confirm the role of RGD- and collagen-binding integrins in MSC attachment to full-thickness cartilage lesions in the more complex *ex vivo* situation, we performed competition and blocking studies in the osteochondral explant system. The RGD peptide was able to compete for about 50% of the attachment of MSC to full-thickness cartilage lesions, whereas the RGD control peptide had no influence (Fig. 4C). Blocking studies with a β1 integrin blocking antibody demonstrated a dose-dependent inhibition of the MSC attachment to defective cartilage with a maximum inhibition of about 90% at 40 μg/mL antibody concentration, whereas an isotype control antibody had no influence on MSC attachment (Fig. 4D). These results fully corroborate our *in vitro* findings and clearly show that attachment of MSC to full-thickness cartilage lesions in our *ex vivo* system is almost entirely β1 integrin-mediated, whereby both RGD- and collagen-binding integrins are involved.

**FIG. 5.**
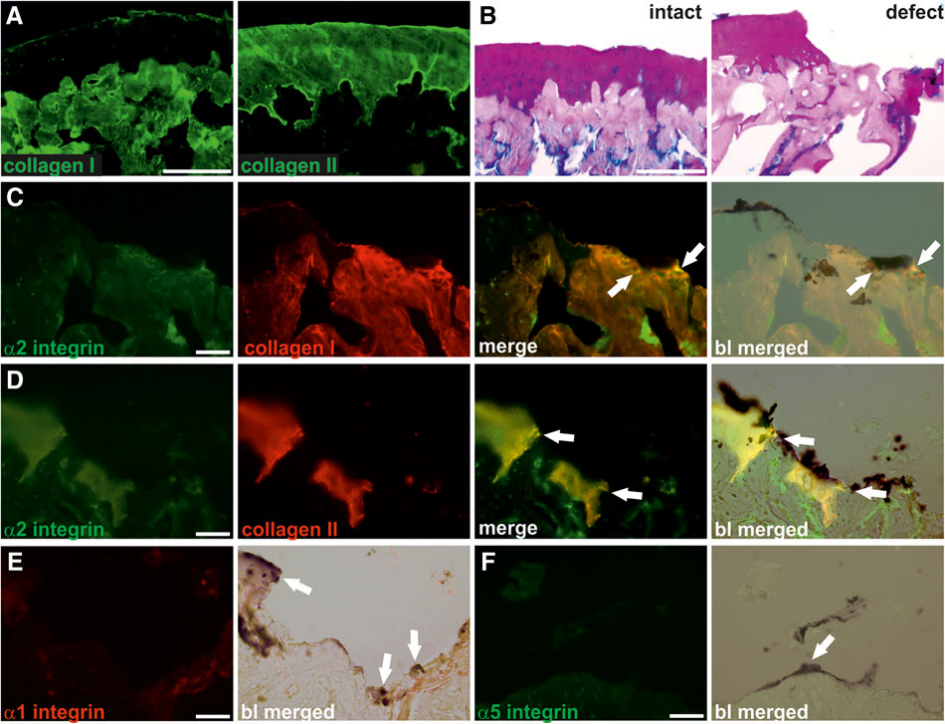
Expression of α2 integrin at MSC attachment sites to bone and cartilage. **(A)** Expression of collagen I and collagen II in cryosections of intact osteochondral explants. Immunofluorescence analysis was performed on cryosections of hPLAP prestained intact osteochondral explants by incubation with polyclonal antibodies against collagen I and collagen II. Collagen I is found in bone tissue, whereas collagen II is exclusively located in cartilage tissue of the intact explant. **(B)** Alcian blue-PAS staining of intact osteochondral explants and explants with a full-thickness defect. Articular cartilage is stained purple, subchondral bone is stained magenta, and proteoglycans are stained blue. **(C, D)** Expression of α2 integrin in osteochondral explants with full-thickness defects. Co-immunofluorescence analysis was performed on cryosections of previously hPLAP-stained and quantified osteochondral explants by parallel incubation with an antibody against α2 integrin **(C, D,** green**)**, and a polyclonal antibody against collagen I **(C,** red**)** and collagen II **(D,** red**)**. α2 integrin and collagen I and II are co-expressed in distinct adjacent zones (merge, yellow, arrows). Merging with bright light (bl) images demonstrated the co-expression of α2 integrin and collagen I at sites where hPLAP-tg MSC attach to subchondral bone **(C,** bl merged, arrows**)**. Bright light-merged images after co-staining of α2 integrin and collagen II revealed co-expressing sites where hPLAP-tg MSC attach to collagen II **(D,** bl merged, arrows**)**. **(E, F)** Expression of α1 and α5 integrin in osteochondral explants with full-thickness defects. Immunofluorescence analysis was performed on hPLAP-prestained cryosections of osteochondral explants by incubation with a monoclonal antibody against α1 integrin **(E)** and a polyclonal anti-α5 integrin antibody **(F)**. Expression of neither α1 nor α5 integrin could be observed at sites where hPLAP-tg MSC attach to the cartilage lesion (bl merged, arrows). Scale bar=50 μm.

To identify potential integrin α-subunits involved in the adhesion of MSC to full-thickness cartilage defects, we performed immunofluorescence analyses (Fig. 5). Staining of cryosections of intact osteochondral explants for collagen I and II confirmed the specificity of the used antibodies. The anti-collagen I antibody clearly stained the bone tissue, whereas collagen II was expressed exclusively in the articular cartilage and calcified cartilage of the epiphysis in intact osteochondral explants (Fig. 5A). The collagen I and II staining pattern was similar to the distribution of bone and cartilage tissue as evidenced by alcian blue-PAS staining (Fig. 5B). Co-immunofluorescence analysis for α2 integrin and collagen I and II demonstrated distinct zones of adjacent expression of α2 integrin and collagen I and II in areas where MSC attached to subchondral bone or hyaline cartilage, respectively (Fig. 5C and D). In contrast, expression of α1 (Fig. 5E) or α5 integrin (Fig. 5F) was not observed in the defects. Taken together, our findings suggest that α2β1 integrin may be one of the key mediators of MSC attachment to bone and cartilage in full-thickness cartilage lesions.

## Discussion

**FIG. 6.**
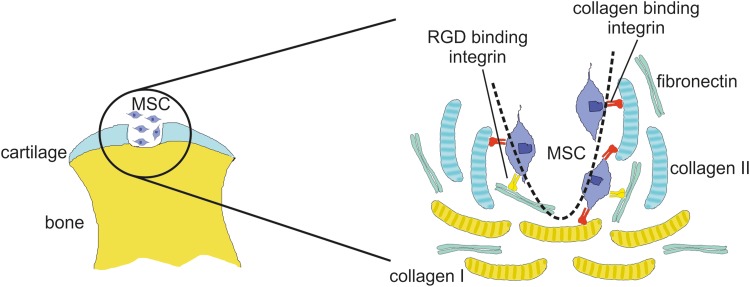
Model for MSC adhesion to articular cartilage lesions. MSC are able to bind to collagen I in bone and to collagen II in cartilage in full-thickness cartilage defects (dashed line) via collagen-binding β1 integrins. In addition, MSC interact via RGD-binding integrins with fibronectin, a major matrix protein present in bone and cartilage.

The key findings in our study were (1) that serum improves MSC attachment to cartilage lesions, (2) that divalent cations are essential for binding of MSC to full-thickness cartilage lesions as well as for MSC binding to collagen I, collagen II, and fibronectin, (3) that β1 integrin blocking antibodies fully block binding of rat MSC to collagen I and collagen II, but only partially inhibit binding to fibronectin, and (4) that RGD peptides partially block the adhesion of MSC to fibronectin *in vitro* and to cartilage lesions *ex vivo*. Thus, our data suggest that both RGD- and collagen-binding integrins are involved in the attachment of MSC to full-thickness articular cartilage lesions. This model is shown in Figure 6. Our study is in line with the findings of Shimaya and co-workers^37^ that magnesium increased adhesion of human synovial MSC to collagen-coated slides in a β1 integrin-dependent fashion *in vitro*, underscoring the importance of β1 integrins for binding of MSC to ECM proteins across species.

The present study has shown that our newly established co-isogeneic *ex vivo* system consisting of MSC isolated from hPLAP-tg donor F344 rats and wild-type F344 osteochondral explants is an excellent tool to study MSC attachment to cartilage lesions. By use of the marker gene *hPLAP*, which is ubiquitously and stably expressed in hPLAP-tg rats and does not influence MSC behavior,^26,27^ we were able to reliably and very efficiently track hPLAP-expressing MSC attached to the wild-type osteochondral explants by histochemical detection methods and subsequent image analysis.

In our *ex vivo* explant system, the near-physiological combination of 1 mM MgCl_2_ with 2.5 mM CaCl_2_ provided the best results in terms of MSC-covered area within the cartilage lesion. Interestingly, calcium had no or even a negative effect on the adhesion of MSC to defective cartilage. Conversely, adhesion of MSC to collagen I, collagen II, and fibronectin *in vitro* was abolished in the presence of 10 mM EDTA. These phenomena have already been described earlier^38,39^ and are consistent with the notion that integrins may be involved in the MSC attachment. Divalent cations are needed for activation of integrins and for keeping them in their active state.^19–21^ ETDA chelates divalent cations, resulting in an inactive state of cell surface integrins.^21^ Thus, our data suggest that the attachment of MSC to ECM proteins accessible in full-thickness cartilage defects is an almost entirely integrin-mediated process.

Cultured MSC express various integrin subunits such as α1, α2, α3, α4, α5, α6, α7, α10, αV, αIIb, β1, β3, β4, and β5.^13–15^ The expression of these integrin subunits on MSC was described earlier on mRNA and protein level. Expression of the subunits α7, α10, αIIb, and β5 has so far only been analyzed by microarray hybridization.^15^ Our *in vitro* cell attachment assays revealed that MSC attach to collagen I, collagen II, and fibronectin, but not to collagen XXII and COMP. Collagen I is the main ECM protein in bone, collagen II is the major matrix molecule in cartilage, and fibronectin is present in high amounts in both tissues. In agreement with the fact that all collagen-binding integrins (α1β1, α2β1, α10β1, and α11β1) contain the β1-subunit,^20^ binding of MSC to collagen I and collagen II *in vitro* was fully blocked by the monoclonal β1 integrin blocking antibody. Furthermore, immunofluorescence analysis on osteochondral explants with cartilage lesions demonstrated expression of α2 integrin in distinct zones where MSC attach to collagen I or collagen II in the lesion, whereas no expression of α1 and α5 integrin could be observed. These distinct zones are likely to be focal adhesion sites that can develop into focal adhesion plaques, and are known as first step of integrin signaling.^20^

The finding that α2β1 integrin may be involved in MSC attachment to cartilage lesions explains the strong influence of magnesium and the lacking influence of calcium in our *ex vivo* attachment experiments, because α2 integrin needs the presence of magnesium rather than calcium to bind to collagens.^41,42^ In addition, β1 integrins were also shown to be essential for MSC attachment and survival in biodegradable polymers.^18^ In contrast to collagens, fibronectin interacts with RGD-binding integrins (e.g., α5β1, αVβ1, αVβ3, αVβ5, and αIIbβ3).^20^ It is interesting to note in this context that the attachment of MSC to fibronectin could not be fully inhibited by the β1 integrin blocking antibody in our experiments, a finding which may indicate that additional RGD-binding β-subunits are involved in the attachment to fibronectin. Because we were unable to detect expression of α5 integrin in immunofluorescence analysis, it is tempting to speculate that αV integrins may mediate the attachment of MSC to fibronectin. Clearly, more work needs to be done to fully characterize all integrins involved in the attachment of MSC to cartilage lesions.

MSC for cell-based therapies are usually suspended in saline,^7^ hyaluronic acid,^5,8^ or serum^6,10^ prior to injection. Our study demonstrated that the addition of serum to the cell suspension provided the best results in terms of MSC attachment to full-thickness cartilage lesions, when compared with plasma, bone marrow plasma, hyaluronic acid, or saline. The difference between serum and plasma might be explained by the fact that EDTA was used to prevent blood coagulation during harvesting of plasma samples. Serum is composed of numerous components, and it is currently unknown which specific serum proteins are able to modulate *ex vivo* and *in vivo* MSC attachment. Clearly, more work has to be done to characterize the individual factors present in serum that improve MSC attachment to cartilage lesions. It is obvious that this knowledge could be exploited to further refine MSC-based therapies.

In conclusion, our study demonstrates the crucial function of β1 integrins for the attachment of MSC to ECM proteins accessible to binding in full-thickness cartilage defects. More experimentation is required to exactly define the nature of the individual α-subunits, and possibly additional β-subunits involved in the attachment process of MSC to ECM proteins present in bone and cartilage. A better understanding of the molecular mechanisms involved in MSC adhesion may lead to significant improvements in MSC-based therapies for osteochondral defect regeneration.

## Abbreviations Used

BCIP: 3-bromo-4-chloro-3-indolyl phosphate
BSA: bovine serum albumin
COMP: cartilage oligomeric matrix protein
ECM: extracellular matrix
FBS: fetal bovine serum
hPLAP: human placental alkaline phosphatase
MEM: minimum essential medium
MSC: mesenchymal stem cells
NBT: nitrotetrazolium blue chloride
OA: osteoarthritis
RT: room temperature
SNK: Student–Newman–Keuls
